# Skeletal muscle tissue engineering: best bet or black beast?

**DOI:** 10.3389/fphys.2014.00255

**Published:** 2014-07-04

**Authors:** Barbara Perniconi, Dario Coletti

**Affiliations:** ^1^Biology of Adaptation and Aging (B2A), Université Pierre et Marie Curie Paris 6Paris, France; ^2^Department of Anatomical, Histological, Forensic Sciences and Hortopedics, Sapienza University of RomeRome, Italy; ^3^Interuniversity Institute of MyologyRome, Italy

**Keywords:** skeletal muscle, stapedius muscle, tissue engineering, 3d cell cultures, regenerative medicine

Skeletal muscle possesses a remarkable self-repair capacity whose underlying mechanisms have been thoroughly investigated (Vandenburgh et al., 1988; De Arcangelis et al., 2003; Musaro et al., 2007; Moresi et al., 2008, 2009) with a view to stimulating *in situ* regeneration. It is, however, unable to restore volumetric tissue loss as a consequence of trauma, congenital defects, ablation, or denervation. This is the rationale behind the creation of new skeletal muscle through tissue engineering (TE). While most review articles announce that skeletal muscle TE is advancing and is readily translatable, it seems clear that engineered skeletal muscle is still lagging far behind other tissues if placed within a clinical practice context. Why is it not yet possible to transplant off-the-shelf, functional muscles into patients?

Major difficulties encountered in skeletal muscle TE on the whole organ scale are currently being addressed. These include the following: the large size of human organs has been overcome by studies on rabbit, dogs and humans (Badylak et al., 1998; Rossi et al., 2010; Badylak et al., 2013); numerous myogenic cell populations of muscle and non-muscle origin (Rossi et al., 2010) are now available as cell sources; scaffolds with a specific 3D orientation are obtained through organ decellularization, freeze drying and electrospinning of synthetic or natural materials (Klumpp et al., 2010); function has been proven for several of these engineered muscle constructs (Mudera et al., 2010; Carosio et al., 2013). Even some of the interactions between muscle and other organs have been addressed: the muscular-tendinous junction can be restored by suturing the residual tendon proximal extremity of muscle-derived acellular scaffolds to the host tendon (Perniconi et al., 2011); the vascular bed can be reconstructed in striated muscles (Koffler et al., 2011; Carosio et al., 2013), particularly if the elegant approach proposed by Ott et al. for decellularized heart is applied (Ott et al., 2008).

Muscles, however, remain very challenging organs to rebuild. Muscle hierarchic architecture and heterogeneous cell composition have not yet been sufficiently investigated by either *in vitro* or *in vivo* studies. Moreover, innervation by the somato-motor system has not been addressed at all. We believe that the latter issue will be the hardest to address; since we are still unable to induce normal re-innervation following motor neuron injury, the successful innervation of a new, pre-built organ transplanted *in vivo* is unlikely to be straightforward. Lastly, even some basic aspects of muscle TE, such as graft bioactivity and integration, which are often claimed to be established, are actually still poorly understood; for instance, the novel idea of cryptic peptides released by the extra-cellular matrix (ECM) while it is being biodegraded and remodeled (Grayson et al., 2009) opens new avenues for the exploration of ECM component bioactivity. This need to gain a better knowledge of the properties of the ECM and bioactive molecules used in TE applications is indeed the rationale of dedicated publications, such as this Frontiers in Muscle Physiology Special Issue.

While obtaining a fully functional, innervated human skeletal muscle from TE that may be used for clinical purposes remains a long-term goal, here we propose two applications for engineered muscle that are no less ambitious and that may be achieved and exploited in the short term. As stated by Grayson et al., the biomimetic effort being made within the context of skeletal muscle TE is mostly aimed at: (a) the creation of functional tissue grafts for regenerative medicine applications *in vivo*; (b) the generation of experimental models *in vitro* for studies on stem cells, development, and disease (where engineered tissues can serve as advanced 3D models).

- As far as organ replacement *in vivo* is concerned, we expect very specific TE-based interventions, such as the application of muscle flaps and the generation of minute individual muscles, to be immediately successful, whereas the mass production of large muscles involved in chronic and global muscle wasting diseases is less unlikely to be so. One example of organ replacement that is likely to be successful is the *Stapedius*, which is the smallest and weakest skeletal muscle in the human body. It stabilizes the stapes, a very small bone in the inner hear, and is innervated by the facial nerve. Dysfunctions in the *Stapedius* induce hyperacusis or other sound perception defects that are clinically relevant. While the problem of innervation is likely to persist, the engineering and grafting of this small and simple muscle to replace the diseased one (often due to a local defect) appears to be highly feasible.
- As far as *in vitro* studies are concerned, it is self-evident that bidimensional cultures are very limited insofar as the physiological 3D tissue organization they yield is somewhat approximate. Muscle TE was initially designed for *in vitro* studies, when Vandenburgh et al. introduced the 3D cultivation of primary myoblasts in collagen gel and generated contracting muscle tissue *in vitro* for the first time in 1988 (Vandenburgh et al., 1988). A progressive increase in the architectural complexity of ECM and cells in tissue-culture grade constructs is likely to provide adequate experimental models for the *in vitro* study of phenomena that are specific to the *in vivo* situation [e.g., stem cell niche, tissue regeneration, aging (Sharples et al., 2012)].

With regard to the two applications described above, it should be borne in mind that the goals and approaches involved in building engineered muscle tissue may not be the same owing to the significant differences between *in vitro* and *in vivo* TE strategies (reviewed by Rossi et al., 2010).

For all these reasons, we believe that the best bet for skeletal muscle TE is to focus on specific, anatomically defined solutions or on 3D *in vitro* modeling of muscle tissue for basic and applied research. We are confident that we will eventually be able to transform the black beast (i.e., striated muscle tissue engineering) into the best bet (i.e., a successful clinical practice based on engineered muscles). However, for more ambitious muscle TE applications, there may still be a long way to go.

## Conflict of interest statement

The authors declare that the research was conducted in the absence of any commercial or financial relationships that could be construed as a potential conflict of interest.
